# Susceptibility to Disinformation: A Data‐Driven Typology Based on COVID‐19 Hoaxes and Pro‐Russian Propaganda

**DOI:** 10.1002/ijop.70186

**Published:** 2026-02-15

**Authors:** Martina Klicperova‐Baker, Martin Jelinek, Petr Kveton

**Affiliations:** ^1^ Institute of Psychology Czech Academy of Sciences Brno Czech Republic

**Keywords:** COVID‐19, Czech Republic, disinformation, EFA, LPA, Russia, Ukraine

## Abstract

An original dataset based on a national quota sample in the Czech Republic (*n* = 490, *M* = 46.09 y/o, 45.7% women) was used to assess susceptibility to medical (COVID‐19) and political (Russian invasion of Ukraine) disinformation. Susceptibility to disinformation was assessed using 30 items addressing contemporary topics. To identify the latent structure underlying these items, an exploratory factor analysis (principal‐axis factoring with direct oblimin rotation) was conducted. EFA yielded four correlated factors: one specific to COVID‐19 hoaxes/misinformation (F1) and three others pertaining to the political (F2), economic (F3), and moral/ethical (F4) dimensions of the Russian war. In order to identify response patterns, all 30 items from 490 participants were subjected to Latent Profile Analysis (LPA) in which the EFA factors served to interpret the five resulting types: a neutral *No Strong Opinion* type (48%); two disinformation‐resilient types—*Rational Pro‐Ukrainians* (22%) and *Anti‐Russians* (7%); and two disinformation‐susceptible types—*Pro‐Russians* (15%) and the *Generally Disinformed* (9%). The discussion addresses the sizable *No Strong Opinion* type and the correlation between COVID‐19 hoaxes and propaganda disinformation (*r* = 0.47), which supports the ‘monological belief system’ concept. The identified types can be further followed prospectively and retrospectively within an ongoing panel study.

## 1 Introduction

Vulnerability to disinformation has become a pressing challenge for contemporary societies. The rapid expansion of electronic communication has democratised the public sphere and enabled the proliferation of alternative information channels. Somewhat paradoxically, these achievements have not only accelerated the diffusion of misleading and false content but have also endowed it with a superficial sense of legitimacy.

The circulation of false information is not a novel phenomenon; however, its present scope and societal impact are unprecedented (Wardle and Derakhshan 2017; Global Risks 2024). Disinformation has reached such prominence that some have felt compelled, controversially, to describe our times as ‘post‐factual’ or ‘post‐truth’—as if most of humanity had collectively abandoned the pursuit of truth. Disinformation—much of it originating from hostile state actors and amplified by naïve citizens—has the capacity to polarise communities, erode public trust in institutions, and distort processes of collective decision‐making (Lewandowsky et al. 2017; Lazer et al. 2018). Considering these far‐reaching consequences, systematic inquiry into disinformation is of critical scholarly and practical importance.

### 1.1 Russian Information Warfare

Whilst many countries engage in propaganda competition, the Russian strategy of hybrid warfare—combining conventional and unconventional tactics (Hoffman 2007)—has proven particularly effective. Its disinformation campaigns were operating long before the full‐scale invasion of Ukraine in 2022. For instance, Smoleňová (2015) highlighted disinformation flows that systematically propagated anti‐Western, anti‐American, and anti‐Ukrainian narratives in line with the ‘divide and conquer’ strategy whilst simultaneously advancing pro‐Russian propaganda. She cited Michal Kubal, head of the Foreign News Department at Czech Television, who noted the Russian efforts to undermine public trust: ‘You don't have to trust the Kremlin; just don't trust anybody.’

Hybrid warfare has intensified and become increasingly sophisticated since then. The European Council, amongst others, has noted extensive pro‐Kremlin disinformation activities (European Council 2025; Győri et al. 2022), which have propagated false claims denying the existence of the Ukrainian nation, alleging Ukraine–U.S. bioweapon laboratories, portraying Ukraine as inherently ‘Nazi,’ and suggesting that it posed a threat of invading Russia. Wenzel et al. (2024) provided evidence that Russian disinformation penetrates Central Europe with varying degrees of effectiveness and assessed Czech vulnerability to hostile propaganda as ‘medium,’ situated between a Slovak ‘high’ and a Polish ‘low.’

### 1.2 Political Propaganda and COVID‐19 Disinformation

Consistent with the logic of hybrid warfare, Russian efforts have not been confined to overt political propaganda but have extended to any topic capable of generating confusion or polarisation within adversary states. During the COVID‐19 pandemic, these operations included the dissemination of conspiracy theories, documented from the earliest stages of the outbreak not only by foreign intelligence agencies but also by scholars and journalists. For example, the BBC (2020) reported that Russian state television promoted conspiracy theories during primetime news broadcasts. Subsequent analyses indicated that COVID‐19 and Russian war propaganda shared overlapping narratives and dissemination channels (e.g., Kovacheva 2023), even though the former relied primarily on written formats whilst the latter predominantly employed audiovisual ones (del Sánchez Vas and Tuñón Navarro 2024).

### 1.3 The Monological Belief System

The literature provides substantial evidence of a widespread tendency to endorse multiple conspiracy theories, even when these theories are mutually contradictory (Myslivec 2024). This phenomenon, first described by Goertzel (1994), is conceptualised as a rigid and self‐reinforcing *monological belief system*—in contrast to the open and critical *dialogical belief system*. Recent research has further demonstrated strong correlations between belief in COVID‐19 disinformation and susceptibility to pro‐Russian propaganda, both internationally and within the Czech Republic (Grygarová et al. 2025; Myslivec 2024).

### 1.4 Towards a Typology of Psychological Susceptibility to Disinformation

The psychology of disinformation has developed rapidly over the past decade, producing extensive evidence on the cognitive, emotional, motivational, and social factors underlying individual differences in susceptibility to false or misleading information. However, most studies focus on isolated predictors rather than identifying integrated psychological profiles or latent types of individuals who share similar dispositions towards disinformation.

#### 1.4.1 From Vulnerability and Resilience to Profiles

Two major lines of research dominate the field. One emphasises *vulnerability*, focusing on cognitive and motivational biases that increase susceptibility to disinformation—such as media illiteracy, epistemic and existential insecurity, or moral absolutism (Douglas et al. 2019; Jones‐Jang et al. 2021; Leone et al. 2019). The other concentrates on *resilience*, highlighting protective traits such as analytical reasoning, open‐mindedness, or trust in institutions (Lewandowsky et al. 2017; Roozenbeek et al. 2022).

Although both perspectives offer valuable and compatible insights, they typically treat predictors as independent dimensions. Consequently, the field presents a fragmented picture: we know which cognitive and social factors matter, but we know far less about how they co‐occur within individuals. Existing research seldom examines how combinations of reasoning style, moral orientation, trust, and ideology cluster together to form distinct patterns of susceptibility or resistance to disinformation.

### 1.5 Gaps in Knowledge: The Neglected Typological Perspective

Person‐centred typological and latent‐profile approaches are well established in other areas of psychology, including personality (Gerlach et al. 2018), coping with stress (Folkman and Lazarus 1980), and political psychology (Klicperova‐Baker and Kostal 2015). These methods provide insights grounded in actual individuals rather than abstract variables. Only a few recent studies have applied this approach to disinformation, segmenting audiences according to their susceptibility profiles. However, these studies are typically monothematic—focusing primarily on willingness to take COVID‐19 vaccines and trust in science (Agley and Xiao 2021; Bruder and Kunert 2022)—and thus offer only a limited view of broader susceptibility patterns.

#### 1.5.1 Recapitulation of the Rationale for the Present Approach

First, we opt for a person‐centred typological approach, which can reveal complex configurations and patterns of susceptibility that remain invisible in variable‐centred analyses. Second, whilst existing research has primarily addressed vulnerability and resilience as opposite ends of the same continuum, our approach also accounts for individuals situated in between—who may represent a substantial portion of the population. Third, our study takes into account more than one domain of disinformation—encompassing both medical and political contexts. Fourth, once established, typologies may be related to additional attitudinal or behavioural outcomes, offering an even more integrative understanding of how different psychological dispositions co‐occur and shape public responses to information in times of uncertainty and crisis.

The present study addresses these gaps by (a) focusing on a diverse set of disinformation items—both medical and political—administered repeatedly at times of topical relevance; (b) including all individuals from a large national sample; (c) developing an empirically derived typology using exploratory factor analysis (EFA) and latent profile analysis (LPA); and (d) establishing a categorisation of more or less disinformed types that can, in addition, be followed prospectively and retrospectively as part of an ongoing panel study.

## 2 The Current Study

### 2.1 The Czech Panel Study

As soon as the COVID‐19 epidemic became a serious problem, we launched a panel study addressing relevant psychological and political issues. The study continued even after Russia invaded Ukraine. Over time, sufficient data accumulated to allow pooling heterogeneous, topic‐diverse items that capture susceptibility to misinformation more as a stable trait than a transient state. This approach aligns with recent psychometric models that conceptualise misinformation discernment as a latent disposition. These models employ exploratory methods to uncover lower‐ and higher‐order dimensions across heterogeneous domains such as health and politics (e.g., Maertens et al. 2024).

### 2.2 Primary Aims

The primary aims of the study were:

- to assess the vulnerability of a national sample to medical and political disinformation and to identify the latent structure of related attitudes using EFA.
- to examine associations between susceptibility to disinformation across different domains (medical and ideological).
- to develop a typology of respondent groups differing in their attitudinal patterns towards disinformation, using LPA, and to compare these types across demographic variables and indicators of democratic orientation.

## 3 Materials and Methods

### 3.1 Participants and Data Collection

This study utilised the original dataset derived from a panel study conducted on a Czech national quota sample (*n* = 490, 45.7% women). Respondents were repeatedly—at real‐time crises—queried about disinformation concerning COVID‐19 and Russian political propaganda. The data were collected by an established opinion poll agency, Median, using computer‐assisted web interviewing (CAWI). Respondents received a modest financial reward (an equivalent of $1 or $2) for completing the survey. The age ranged from 18 to 69 years (*M* = 46.09, SD = 13.40). Regarding education, 181 participants had only basic education, 179 completed high school education with a diploma, and 130 held a university degree.

### 3.2 Measures

*Disinformation about COVID‐19 was assessed by* 12 items (C‐1 to C‐12) ranging from popular misperceptions to irrational hoaxes; their exact wording is presented in Table 1. They were administered *in two sets*: items C‐1 to C‐10 in March 2021 and items C‐11 and C‐12 from May 24 to June 15, 2022. The first set was introduced by an instruction: ‘People in our country and abroad say various things about the COVID pandemic. Which of the following statements do you agree with, and to what degree? Please use the following scale: with a response scale of 1 = *I do not agree at all* to 7 = *I fully agree*.’ The second set was prompted by the request: ‘Please mark for each of the following statements how likely it is in your opinion that the given statement is true.’ The responses were marked on a percentage scale with 10% increments and verbal labels. The scale ranged from 0% = *absolutely out of the question* to 100% = *absolutely sure*.

**TABLE 1:** List of statements about the COVID‐19 pandemic and the war in Ukraine.

Attitudes to the COVID‐19 pandemic	
C‐1	When it comes to the risk of contracting COVID, any mask is better than no mask.
C‐2	A person who does not have COVID symptoms himself cannot infect others.
C‐3	COVID‐19 is no more dangerous than the common flu
C‐4	COVID vaccines were made in a hurry and so will have serious side effects that will only become apparent in the future.
C‐5	Reporters, scientists and the government are deliberately withholding important information from us about COVID‐19.
C‐6	Vaccines are not beneficial in any way, they just bring great profits to the big pharmaceutical companies.
C‐7	The big companies are hiding the fact that COVID can be treated with cheap traditional natural remedies.
C‐8	Bill Gates is sponsoring COVID‐19 vaccines to spread microchips to humans.
C‐9	The COVID‐19 pandemic is largely influenced by the gradual expansion of the 5G mobile network.
C‐10	The COVID‐19 pandemic is artificially induced to spread fear and subsequent population control
C‐11	The primary function of the COVID vaccine is to serve the interests of the pharmaceutical industry.
C‐12	Vaccination against COVID is an irresponsible experiment on humans.

Attitudes to the war in Ukraine	
U‐1	The military invasion of Ukraine by Russia has been described by Western statesmen and representatives of the Czech Republic as an unjustifiable act of aggression. Do you agree with this statement?
U‐2	Russia and its invasion of Ukraine are to blame for the high cost we are now experiencing (energy, food).
U‐3	Ukraine is nationalistic and has discriminated against the Russian‐speaking population.
U‐4	Ukraine is fascist and was planning the genocide/murder of the Russian speaking population.
U‐5	Ukraine was preparing an attack on Russia.
U‐6	Without Russia there will not be enough energy (gas) for the winter.
U‐7	It is necessary to come to an agreement with Russia directly to resume gas supplies.
U‐8	Anti‐Russian sanctions ultimately hurt us more than Russia.
U‐9	It would be better to lift the sanctions against Russia already.
U‐10	Our politicians are helping the Ukrainians more than our own people.
U‐11	Instead of looking after Ukrainian immigrants, our government should be looking after our own people.
U‐12	Ukraine should be part of Russia.
U‐13	In any conflict, and therefore now in Ukraine, the blame lies on both sides.
U‐14	Ukrainians are an independent nation and so have the right to apply to the EU and NATO regardless of Russia's wishes.
U‐15	We should all make sacrifices to help Ukraine.
U‐16	Almost all Russians bear responsibility for what is happening in Ukraine now.
U‐17	The conflict in Ukraine is the fault of Western countries (the USA and/or the European Union).
U‐18	Most Russians are guilty of what is now happening in Ukraine.

*Agreement with political propaganda regarding the war in Ukraine* was measured by 18 items (U‐1 to U‐18) ranging from resolute pro‐Ukrainian support to pro‐Russian propaganda; the exact wording is given in Table 1. The items were administered in three sets, between April and November 2022. Responses to the U‐1 question were marked on a 5‐point Likert scale ranging from 1 = *I definitely disagree* to 5 = *I certainly agree*. The U‐2 to U‐5 items were administered using the same instruction and percentage response scale as were used for COVID items C‐11 and C‐12 described above. The rest of the Ukraine‐related items (U‐6 to U‐18) were introduced by the instruction: ‘People differ in their opinions on various phenomena and behaviour in our country and abroad. Please mark how much you agree with the following statements.’ Again, a 7‐point Likert scale was used, ranging from 1 = *I do not agree at all* to 7 = *I fully agree*.

*The importance of democracy* was assessed using the question ‘How important is it for you to live in a country that is governed democratically?’ administered in November 2022. The response scale ranged from 1 = *Not important at all* to 10 = *Extremely important*.

### 3.3 Analytical Procedures

To maximise the validity and reliability of our measurement of disinformation susceptibility, we combined all relevant items collected across several panel waves. Although these items were administered at different points in time and referred to distinct topical contexts (COVID‐19 disinformation and pro‐Russian propaganda), they were designed to capture the same underlying disposition—individuals' general susceptibility to disinformation. We therefore treated them as heterogeneous but conceptually related indicators of a single latent construct, rather than as repeated time‐specific measurements. The EFA supported this approach, as items from different waves loaded together on common factors, indicating that the structure was driven by thematic rather than temporal similarity. This approach likely increased construct validity and reliability (cf. Proust‐Lima et al. 2022; Zhan et al. 2017).

The first step involved EFA, exploring the underlying factor structure of 30 items (12 items related to COVID and 18 items related to the war in Ukraine). EFA was conducted using principal‐axis factoring with oblimin rotation. The optimal number of EFA factors was determined on the basis of four criteria: Comparison Data, Optimal Coordinates, Parallel Analysis, and Velicer's MAP. The factors extracted by the EFA were subsequently used as the dimensions for the latent profile analysis.

The second step involved LPA. The EFA factor scores (using Thurstone's regression‐based weights method) were subsequently analysed using LPA in the R mclust package (Scrucca et al. 2016) to create a typology of individuals. The adequacy of model selection and the number of components in this analysis were determined using BIC and ICL indices, which are recommended by Scrucca et al. (2016). The five identified LPA groups were compared in terms of gender, age, education, and *perceived importance of democracy* using Pearson's chi‐squared test.

## 4 Results

### 4.1 Exploratory Factor Analysis (EFA)

The levels of medical and political disinformation were assessed using 30 items (12 related to COVID‐19 and 18 to the war in Ukraine). To identify the latent structure underlying these items, an exploratory factor analysis (principal‐axis factoring with direct oblimin rotation) was conducted. The KMO index value (0.95) and Bartlett's test of sphericity (*K* ^2^ = 1259.10, df = 29, *p* < 0.001) suggested that the dataset is suitable for factor analysis. All four indices focused on determining the optimal number of factors (see Analytical Procedures section) consistently indicated the appropriateness of a four‐factor solution for capturing the underlying structure of relationships within the correlation matrix. Table 2 shows the *four resulting factors* along with the standardised factor loadings within each factor. As the indicators of attitudes towards the war in Ukraine and COVID‐19 were chosen to represent a wide spectrum of perspectives, items with cross‐loadings were retained in the solution. Their influence was accounted for in the regression factor scores method (see Analytical Procedures section).

**TABLE 2:** Standardised factor loadings (pattern matrix) of attitudes to the COVID‐19 pandemic and to the war in Ukraine.

	Single COVID factor	Three Russian‐War‐in‐Ukraine factors		
	COVID misinformation and Hoaxes beliefs [COVID Hoaxes]	Political: Pro‐Russian Propaganda beliefs [Pro‐Russian politics]	Economic: Anti‐Ukrainian self‐centeredness [Anti‐Ukrainian economy]	Moral: Most Russians' guilt and/or Responsibility [Most‐Russians' guilt]
C‐10	0.**85**	0.02	−0.04	−0.01
C‐6	0.**82**	0.04	0.05	0.02
C‐7	0.**78**	−0.07	0.07	−0.08
C‐5	0.**77**	−0.14	0.12	−0.07
C‐4	0.**73**	−0.12	0.19	0.01
C‐3	0.**71**	0.16	−0.20	0.05
C‐12	0.**63**	0.11	0.12	−0.09
C‐11	0.**61**	0.07	0.23	−0.11
C‐8	0.**51**	0.40	−0.22	0.14
C‐9	0.**44**	0.38	−0.13	0.11
C‐1	**−0.41**	−0.19	0.20	−0.02
C‐2	0.**40**	0.11	−0.03	0.14
U‐1	−0.02	**−0.76**	0.02	0.08
U‐12	0.03	0.**72**	0.07	0.12
U‐3	0.02	0.**69**	0.09	−0.12
U‐4	0.08	0.**69**	0.05	−0.09
U‐14	0.00	**−0.68**	0.02	0.11
U‐9	−0.02	0.**62**	0.31	−0.07
U‐5	0.13	0.**61**	0.07	−0.02
U‐17	0.06	0.**54**	0.31	−0.06
U‐2	0.05	**−0.49**	−0.03	0.16
U‐10	0.13	0.06	0.**79**	0.05
U‐11	0.15	0.08	0.**75**	0.02
U‐8	0.03	0.24	0.**62**	−0.10
U‐13	0.04	0.18	0.**48**	−0.08
U‐7	0.03	0.**47**	0.**47**	−0.03
U‐15	−0.14	−0.08	**−0.42**	0.23
U‐6	−0.07	0.**41**	0.**42**	−0.05
U‐16	−0.02	0.03	0.01	**0.84**
U‐18	−0.03	−0.09	0.08	**0.84**

*Note:* Factor loadings higher than 0.40 are in bold. For item wordings, see Table 1. The factor abbreviations used in the test are in square brackets.

*The resulting factor structure was coherent and theoretically meaningful*. All 12 COVID‐19‐related items are grouped into a single (F1) factor (SS loading = 5.79, proportion variance 0.19, which is labelled ‘COVID Hoaxes’). In contrast, the internally heterogeneous attitudes and beliefs towards the war in Ukraine separated themselves into three factors: (F2) *political* legitimacy of Russian aggression vs. legitimacy of Ukrainian resistance (SS loading = 5.76, 19% variance, labelled ‘Pro‐Russian Politics’); (F3) *economic* grievances criticising that the Czech government helps Ukrainians more than ‘our own people’ and/or criticisms of the anti‐Russian economic sanctions vs. altruistic help to Ukraine (SS loading = 3.68, proportion variance 0.12, labelled ‘Anti‐Ukrainian Economy’ or ‘Anti‐UA Economy’ for short); and finally, (F4) *moral* responsibility or guilt of all Russians for what happens in Ukraine vs. no responsibility and guilt (SS loading = 1.88, proportion variance 0.06, labelled ‘Most Russians' Guilt’).

*All four factors were closely interrelated* (Table 3). The strongest correlation was observed between the ‘Pro‐Russian Politics’ and ‘Anti‐Ukrainian Economy’ factors (*r* = 0.53). This is consistent with theoretical expectations given the ideological alignment of these orientations. The second strongest correlation (*r* = 0.47) was observed between belief in ‘COVID Hoaxes’ and susceptibility to ‘Pro‐Russian Politics.’ Although a significant correlation was expected—consistent with the monological belief‐system framework—we were nevertheless surprised by its magnitude. Figure 2 shows that this association is driven primarily by the widespread *rejection of both* ‘COVID‐19 Hoaxes’ and ‘Pro‐Russian Politics,’ rather than by the concurrent acceptance of both.

**TABLE 3:** Inter‐factor correlations.

	Pro‐Russian politics	Anti‐Ukrainian economy	Most Russians' guilt
COVID Hoaxes	0.47	0.36	−0.13
Pro‐Russian politics		0.53	−0.33
Anti‐Ukrainian economy			−0.30

*Note:* The estimates are Pearson product–moment correlations of the regression factor scores.

### 4.2 Latent Profile Analysis (LPA)

In order to identify response patterns based on their attitudes to COVID‐19 hoaxes and the Russian war, all 30 items from 490 participants were subjected to LPA. The four identified EFA factors (F1 to F4) served to interpret the five resulting types. We considered three models with the highest BIC index values to interpret the LPA results. All identified models belonged to so‐called VVI models characterised by varying volume and shape (and undefined orientation). The three models differed in the number of groups identified: five (BIC = −4618.31), six (BIC = −4603.35), or eight (BIC = −4639.78) groups. The ICL index, which accounts for entropy, prioritises models with 5 groups (ICL_5_ = −4738.25; ICL_6_ = −4755.10; ICL_8_ = −4810.29). Since entropy measures the degree of overlap between groups and ICL favours solutions with minimal overlap (Scrucca et al. 2016), the five‐group model was selected as being the most interpretable.

**FIGURE 1:**
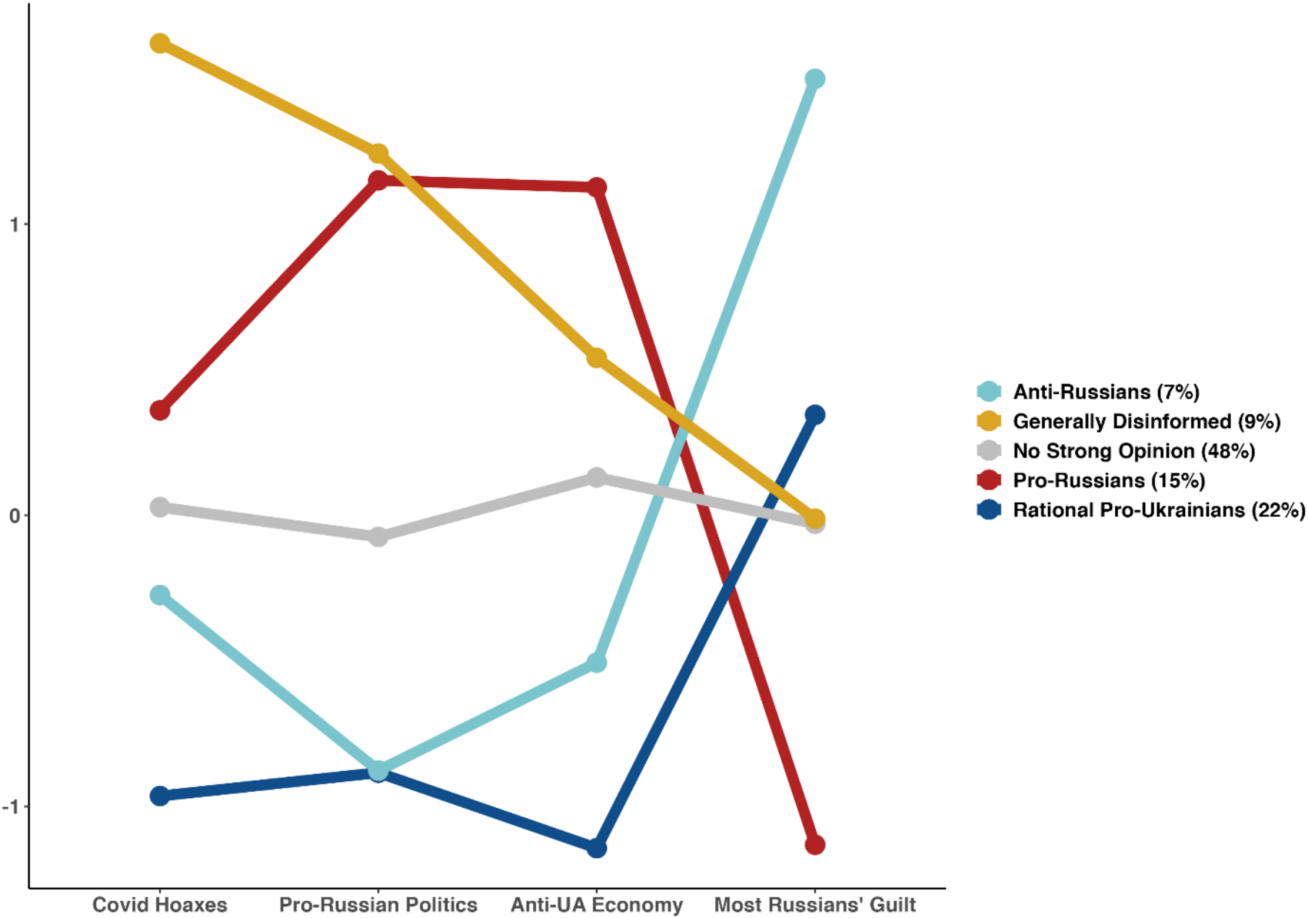
Latent profile analysis results‐group mean values (*n* = 490). Vertical axis shows standardised factor scores on four factor analysis dimensions.

The five LPA‐derived profiles correspond to distinct political ideologies (Figure 1). They vary in perceptions of reality (disinformation) and across political, economic, and moral dimensions of the Ukraine–Russia conflict. Using mean factor scores, the groups can be interpreted as follows:

- *No Strong Opinion* (47.8%, *n* = 234). The largest group, almost half the sample, exhibits a centrist, middle‐of‐the‐road profile. These respondents appear either politically disengaged or unwilling to disclose firm opinions for a variety of reasons.
- *Rational Pro‐Ukrainians* (21.8%, *n* = 107). The second largest profile consistently rejects disinformation (both ‘COVID Hoaxes’ and ‘Pro‐Russian Politics’) and also rejects ‘Anti‐Ukrainian Economy’ attitudes. Their ambivalence about attributing collective guilt to Russians is compatible with a liberal reluctance to endorse collective responsibility. Additional responses (Figure 3) indicate that this profile is firmly pro‐democratic.
- *Anti‐Russians* (6.7%, *n* = 33). Similar to the previous profile, Anti‐Russians also resolutely reject ‘Pro‐Russian Politics’; however, they overwhelmingly attribute responsibility/guilt for the violence in Ukraine to most Russians; they have the highest score on the ‘Most Russians' Guilt’ dimension. Anti‐Russians also differ from Rational Pro‐Ukrainians by exhibiting less resolute rejection of both ‘Covid Hoaxes’ and ‘Anti‐Ukrainian Economy.’ Additional responses, shown in Figure 3, indicate that this profile is pro‐democratic, albeit not as firmly as the Rational Pro‐Ukrainians group.
- *Pro‐Russians* (15.1%, *n* = 74). This group forms a near mirror image of the Rational Pro‐Ukrainians and Anti‐Russian groups. Pro‐Russians strongly reject claims of widespread Russian guilt or responsibility and record the highest scores on ‘Anti‐Ukrainian Economy’—expressing intense opposition to assistance for Ukrainians, which is perceived as coming at the expense of ‘our own people.’ Overall, they conform to ‘Pro‐Russian Politics,’ as does the final group.
- *Generally Disinformed* (8.6%, *n* = 42). Members of this profile strongly accept both ‘COVID Hoaxes’ and ‘Pro‐Russian Politics’ narratives. They present as archetypal anti‐system rebels, showing broad susceptibility to harmful disinformation.

**FIGURE 2:**
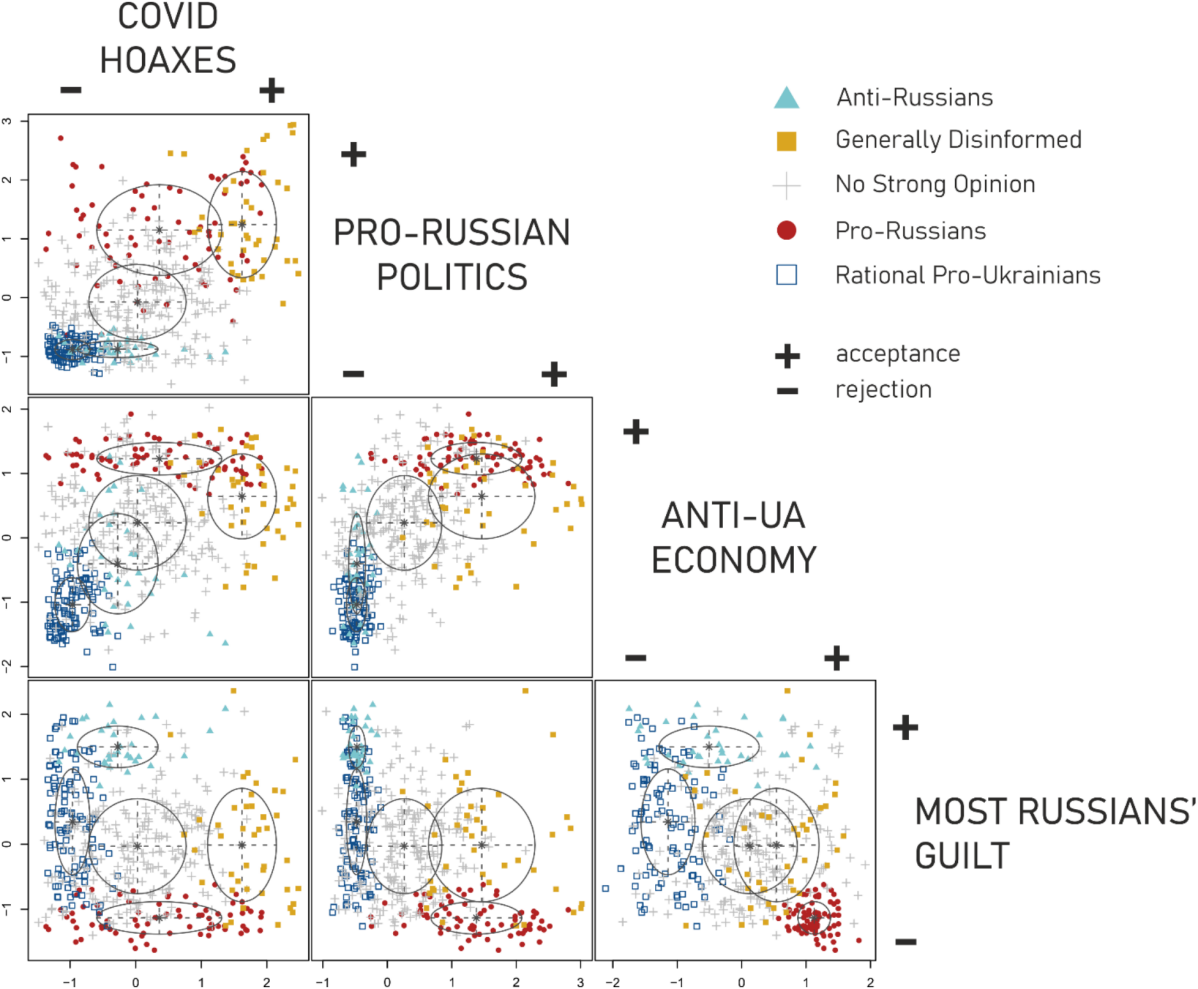
Latent profile analysis results‐classification of individual respondents. Ellipses represent covariances. Both axes show standardised factor scores. Colours indicate LPA‐group membership.

Statistically significant differences between groups were observed on all dimensions represented by the factor scores (all *F*s(4, 485) > 90.00, *p* < 0.001). In the case of the ‘COVID Hoaxes’ dimension, all groups differed from each other except for the *Anti‐Russians* and *No Strong Opinion* pair (based on Tukey post hoc tests). Regarding the ‘Pro‐Russian Politics’ dimension, there were no statistically significant differences between *Rational Pro‐Ukrainians* and *Anti‐Russians* groups, nor between the *Pro‐Russians* and the *Generally Disinformed* groups on the other end of the opinion spectrum. On the ‘Anti‐Ukrainian Economy’ dimension, all groups differed from each other. On the last, moral ‘Most Russians' Guilt’ dimension, only the *No Strong Opinion* vs. *Generally Disinformed* and the *Generally Disinformed* vs. *Rational Pro‐Ukrainians* groups did not differ significantly. Figure 2 provides a visual representation of each respondent's answers across all factor pairings where colours indicate LPA‐group membership.

#### 4.2.1 Demographic Characteristics of the LPA Groups

Groups differed significantly in age, education, and gender. *Rational Pro‐Ukrainians* were the youngest; *Pro‐Russians* and *Anti‐Russians* were older (*M* > 50 years; *F*(4, 485) = 5.90, *p* < 0.01). Higher education was typical of the *Rational Pro‐Ukrainians* but rare amongst the *Pro‐Russians* and entirely absent in the *Generally Disinformed* group, *χ* ^2^(8) = 59.11, *p* < 0.01, Cramer's *V* = 0.25. Men were overrepresented amongst *Rational Pro‐Ukrainians* and *Anti‐Russians*, whilst women were overrepresented in the *Generally Disinformed* group, *χ* ^2^(4) = 15.93, *p* < 0.01, Cramer's *V* = 0.18. Descriptive statistics are presented in Table 4.

**TABLE 4:** Gender, education and age of LPA types.

	No strong opinion	Rational Pro‐Ukrainians	Anti‐Russians	Pro‐Russians	Generally disinformed
Age					
Mean (SD)	45.7 (13.5)	42.3 (13.7)	50.0 (12.4)	51.3 (11.9)	45.9 (12.7)
Gender					
Female	50.0	33.6	33.3	44.6	64.3
Male	50.0	66.4	66.7	55.4	35.7
Education					
College/University	23.9	48.6	36.4	13.5	0.00
High school with diploma	39.3	30.8	30.3	40.5	33.3
Basic school without diploma	36.8	20.6	33.3	45.9	66.7

*Note:* The column percentages sum to 100 (subject to rounding error).

#### 4.2.2 Democratic Dimension and the LPA Groups

**FIGURE 3:**
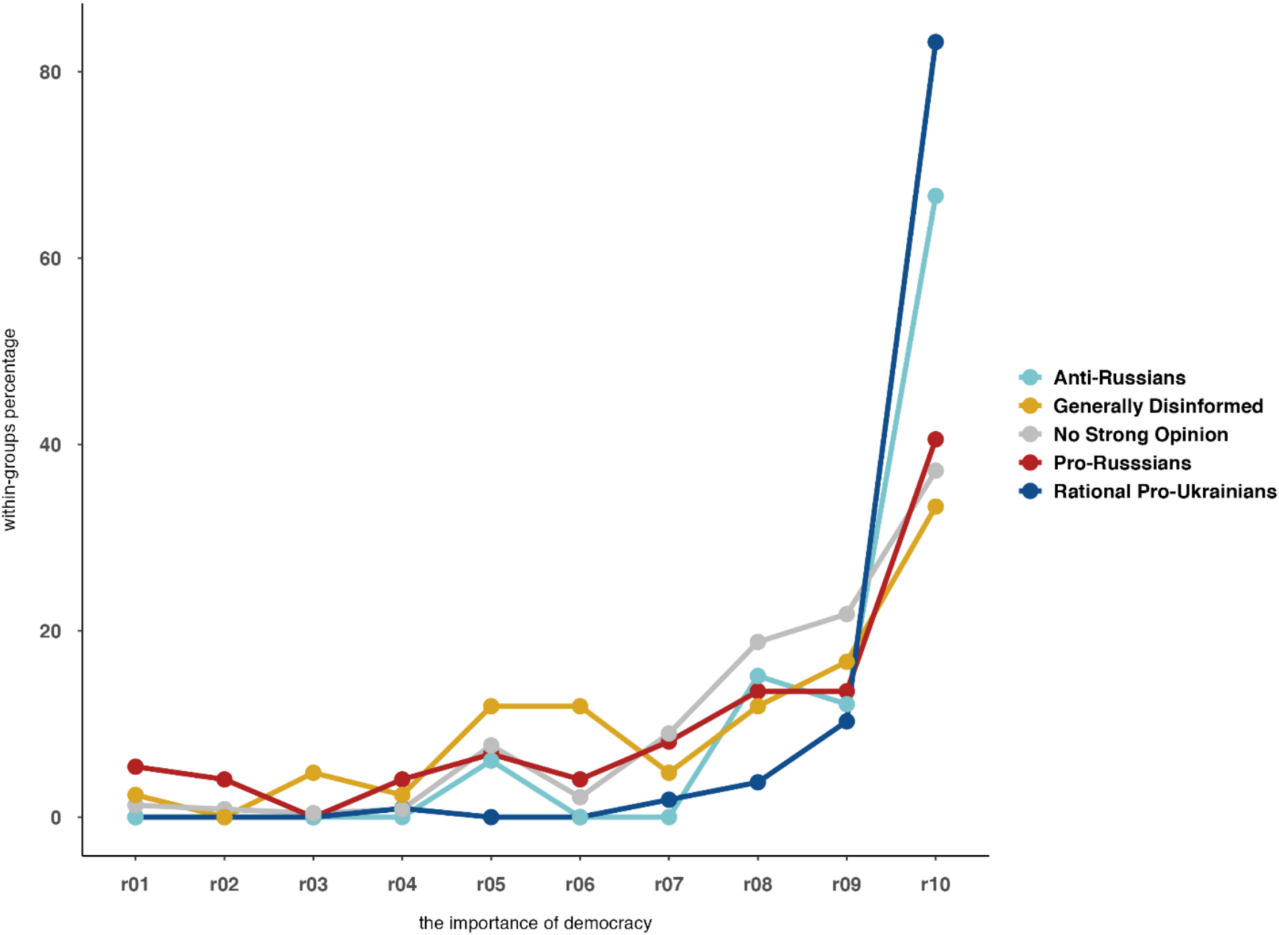
The importance of living in a democratic country according to group membership. The rating scale on the horizontal axis ranges from r01 = *Not important* to r10 = *Extremely important*.

The democratic spirit was assessed with a standard item on the importance of living in a democratic country. Figure 3 shows that, although democracy is valued across all groups, they differ in the proportion who consider living in a democratic system to be ‘extremely important.’ For statistical comparison, the response scale was dichotomized into the maximum response (10) versus all other options. The LPA groups differed significantly in their valuation of democracy, *χ* ^2^(4) = 73.42, *p* < 0.001. Figure 3 indicates that both the *Rational Pro‐Ukrainians* and *Anti‐Russians* groups overwhelmingly agreed that living in a democratic system was ‘extremely important’ (standardised residuals = 5.0 and 1.4, respectively). By contrast, the remaining groups exhibited a less enthusiastic stance (standardised residuals ranging from −1.1 to −2.7). This democratic dimension complements the characterisation of the five profiles: both the *Rational Pro‐Ukrainians* and *Anti‐Russians* groupings appear committed to democracy, with the former somewhat more enthusiastic and marked by liberal concerns (e.g., reluctance to attribute collective guilt to Russians).

## 5 Discussion

### 5.1 The Importance of Studying Disinformation

Disinformation has been likened to a modern epidemic—an infodemic or a digital plague (e.g., Sittig 2023). Moreover, attitudes rooted in disinformation—comprising cognitive, emotional, and conative components—tend to be highly resistant to change and are unlikely to be altered by factual information alone (cf. Porter et al. 2024). Disinformation dissolves social cohesion, undermines trust, and destroys democratic order (cf. Hartz et al. 2025). This danger is particularly acute in the former post‐communist countries and former Soviet satellites (Guasti 2020).

Our study identified a significant number of individuals vulnerable to disinformation in both health and political contexts. Susceptibility to ‘COVID Hoaxes’ and to ‘Pro‐Russian Politics’ correlated strongly (*r* = 0.47), supporting the notion of a domain‐general predisposition to misinformation (Myslivec 2024; Williams et al. 2022). Amongst our respondents, two profiles—*Pro‐Russians* and *Generally Disinformed*—were particularly susceptible to disinformation; together they comprised roughly a quarter of the sample (24%). By contrast, two profiles that appear resilient to disinformation—the *Rational Pro‐Ukrainians* and *Anti‐Russian*s—taken together account for less than one‐third of the sample (29%). At face value, this suggests a small numerical advantage (5%) for the resilient‐to‐disinformation profiles. However, this advantage cannot be relied upon because the largest LPA‐generated group—*No Strong Opinion* (48%)—remains only weakly characterised and should not be considered truly neutral regarding disinformation effects.

### 5.2 Limitations of the Study

This study employed a *non‐probabilistic* quota sample. The non‐random nature of the sample and the potential effects of attrition across measurements should be considered when interpreting the findings, and the prevalence estimates for the LPA‐derived profiles should thus also be treated with caution. Whilst the reported percentages convey an impression of precision, they remain *statistical estimates* rather than measurements—an issue that is particularly pronounced for profiles with smaller sample sizes. The sample was diverse in age, gender, education, and residence, yet it may not fully capture the perspectives of all demographic groups. Some were likely under‐sampled (particularly people who avoid or lack access to communication technologies), whereas others may have been oversampled, such as social media users.

### 5.3 Generalizability

The data were drawn from a national survey conducted in the Czech Republic—a strategically located Central/Eastern European country with a historical legacy shaped by both democratic and totalitarian regimes. The country represents an optimally suited case for studying disinformation because (a) as a former member of the Soviet sphere of influence, it has been significantly exposed to Russian propaganda, and (b) its inhabitants are considered to exhibit a moderate level of susceptibility to disinformation (Wenzel et al. 2024). It is obvious that even within Central Europe, societies differ in their responses to disinformation: Surveys indicate that whereas Hungarians and Slovaks have been shown to display higher susceptibility to Russian disinformation than Czechs, Poles have been more resilient (cf. Brezina et al. 2023; GLOBSEC 2024). Still, despite the contextual differences, the general tendencies and patterns identified in this study are likely relevant internationally. Moreover, in the current global context, where authoritarian and populist movements increasingly rely on disinformation strategies, insights from this study may hold relevance far beyond Central Europe.

### 5.4 Future Research

Despite attempts to frame the current era as ‘post‐truth’ or ‘post‐factual,’ truth is widely recognised as a cornerstone of social life and democratic governance. As Arendt (1967) argued, the deliberate distortion of truth undermines the very conditions of collective trust. Habermas (1984) similarly emphasised that truth‐oriented communication is essential for the functioning of the public sphere. The ongoing erosion of truth and trust is widely regarded as a driver of polarisation and democratic backsliding (Lewandowsky et al. 2017). Thus, safeguarding truth in public discourse is not merely an epistemic ideal but a practical necessity for social cohesion and democratic resilience. A better understanding of disinformation processes is essential for developing effective interventions.

An obvious avenue for further research in this area lies in identifying variables that foster resilience (protective factors) or help better understand vulnerability to disinformation (cf. Douglas and Sutton 2018). However, our study suggests a more specific path. At this stage of our research, we plan to examine more closely the qualitatively distinct types identified in our disinformation typology. We intend to conduct validation studies that will not only confirm the anticipated differences amongst these subtypes but also shed light on why they are differentiated in the first place and how they may continue to evolve. Panel data allow us to address at least some of these questions (cf. Klicperova‐Baker et al. 2026).

Moreover, whilst we have a general understanding of the two extremes—those who are clearly resilient or highly vulnerable—this study draws attention to an often‐overlooked segment of substantial numerical strength that typically remains under the radar. Who constitutes the plurality of respondents with ‘no strong opinion’? To what extent are they disengaged parochials or individuals latently inclined towards socially undesirable attitudes? This grey zone—whether it is largely composed of a silent or passive majority (Almond and Verba 1963) or ambivalent citizens (Lavine et al. 2012)—deserves closer scholarly attention. By exploring this middle ground, future research—including our ongoing panel analyses—may shed further light on the social foundations of resilience and vulnerability to disinformation.

### 5.5 Conclusions

The present study proposed a data‐driven typology of psychological susceptibility to disinformation using data collected in the Czech Republic during two crises—the COVID‐19 pandemic and the Russian invasion of Ukraine. By combining exploratory factor and latent profile analyses, we identified four correlated attitudinal dimensions and five respondent profiles reflecting qualitatively distinct patterns of belief. The results demonstrate that susceptibility to medical and political disinformation is not domain‐specific but instead reflects a broader, domain‐general disposition. The robust correlation between belief in ‘COVID Hoaxes’ and endorsement of ‘Pro‐Russian Politics’ propaganda (*r* = 0.47) lends strong support to the concept of a monological belief system, in which conspiratorial worldviews and propagandistic narratives reinforce each other.

At the societal level, our findings underscore that disinformation is not confined to a small extremist fringe but represents a multi‐layered phenomenon cutting across social and ideological boundaries. Roughly one quarter of respondents fell into the *Pro‐Russian* or *Generally Disinformed* group profiles, displaying consistent susceptibility to false narratives. Yet, a similarly sized minority of *Rational Pro‐Ukrainians* and *Anti‐Russians* groups exhibited resilience, rejecting disinformation and valuing democracy. The largest group—nearly half of the sample—comprised respondents with *No Strong Opinions*, suggesting that indifference or disengagement may constitute a different form of vulnerability. Understanding this ambivalent middle is essential for effective counter‐disinformation strategies, as the stability of democratic systems may depend less on the extremes than on this silent plurality.

Conceptually, this study contributes to the growing effort to map disinformation not merely as a cognitive deficit but as a structured and measurable psychological disposition. Practically, it highlights the importance of integrating both health‐ and security‐related narratives into unified frameworks of misinformation research. Future studies should further validate the typology presented here, investigate its antecedents and longitudinal stability, and explore protective factors that foster resilience to disinformation. In a time when truth itself has become contested, developing empirically grounded typologies such as this one can help identify the psychological fault lines along which democratic societies are most at risk—and perhaps also offer the greatest potential for political renewal.

## Author Contributions

**Martina Klicperova‐Baker:** conception and design, collection of data, drafting the article. **Martin Jelinek:** analysis and interpretation of data; drafting the article. **Petr Kveton:** analysis and interpretation of data.

## Funding

This study was funded by the Johannes Amos Comenius Operational Programme (OP JAC) No. CZ.02.01.01/00/23_025/0008715—(COREMIND) provided by MŠMT; the NPO ‘Systemic Risk Institute’ funded by the European Union—Next Generation EU (Ministry of Education, Youth and Sports, NPO: EXCELES), LX22NPO5101; Strategy AV21/32 Grant ‘Identities in the World of Wars and Crises’ by the Czech Academy of Sciences; and a COVID grant by the Institute of Organic Chemistry and Biochemistry (IOCB), Prague.

## Ethics Statement

All procedures complied with the principles of the Declaration of Helsinki (1964) and its later amendments. Respective waves of the study were approved by the Institutional Ethics Committee of the Czech Academy of Sciences. Moreover, the opinion‐poll agency Median fully complies with applicable EU legislation (including GDPR) and the ICC/ESOMAR Code of Conduct. Only adults were eligible to participate.

## Consent

Informed consent was obtained from all individual participants included in the study and participants were free to withdraw at any time.

## Conflicts of Interest

The authors declare no conflicts of interest.

## Data Availability

Data analyzed in this study are archived in an institutional data repository (https://doi.org/10.14473/CSDA/KLD4TV).
